# Activating discipline specific thinking with adaptive learning: A digital tool to enhance learning in chemistry

**DOI:** 10.1371/journal.pone.0276086

**Published:** 2022-11-15

**Authors:** Paulette Vincent-Ruz, Nathan R. B. Boase

**Affiliations:** 1 Department of Chemistry & Biochemistry, New Mexico State University, Las Cruces, New Mexico, United States of America; 2 Centre for Materials Science, Queensland University of Technology, Brisbane, Queensland, Australia; 3 School of Chemistry and Physics, Queensland University of Technology, Brisbane, Queensland, Australia; University of Idaho, UNITED STATES

## Abstract

In tertiary science education, students are encouraged to engage in discipline specific thinking, to learn their chosen subject. The challenge for educators is engaging all students equitably, despite their educational backgrounds and depth of discipline specific knowledge. Personalising learning in the context of large-scale tertiary courses can only be achieved by using digital technologies. In the context of chemistry education, this project has investigated how an adaptive learning technology can effectively and consistently engage students in discipline specific thinking, by personalising their learning pathway. Adaptive learning has been integrated into a foundational chemistry subject and through quantitative analysis there is empirical evidence to support the benefit adaptive learning has on outcomes, in both the short and long term. This study shows adaptive learning can equitably meet the needs for all students and can lead to improvements in educational behaviour beyond grades. The evidence supports adaptive learning as one critical tool for chemistry educators, and educators in other disciplines of science, to include in their suite of pedagogical strategies to meet the needs of all their students.

## Introduction

Traditional higher education institutions, such as universities and colleges face many critical issues in the contemporary tertiary education landscape. These include increased student cohorts, a requirement to improve student progression and retention rates, and increased competition from other education providers [1–3]. These challenges present problems for university educators, who are faced with a widening diversity of student preparedness for tertiary education and students who may come into college with different intellectual resources [4–7]. Increasing class sizes and the move towards online and blended modes of study has led to less one-on-one time with students, challenging educators to meet the individual experience and needs of their learners.

Students come into the classroom with different prior knowledge for many reasons, including (but not limited to) inequitable access to educational opportunities [5, 6]. Therefore, when it comes to what a learner brings into an experience, it is important that educators focus on malleable factors; constructs that can be shaped and changed through their experience [8]. Furthermore, given the challenges educators face, it is also important to understand not only what the learner brings, but also how the learner’s resources interact with the learning environment that is created.

There is a need to increase our understanding on how to support students’ learning of chemistry in STEM knowledge-centred environments no matter the student’s starting point [9, 10]. This project aimed to embed a digital adaptive learning tool into a university course, as an additional study aid that could provide students with personalised opportunities to learn. It was hypothesised that the personalised and responsive nature of the adaptive technology intervention would enhance student engagement and ultimately enhance academic achievement for students. Specifically, these enhancements would be achieved by increasing student engagement through personalising a student’s learning pathway and by providing rapid and effective feedback. It was hypothesized that by using an adaptive approach to "meet students where they are at" would allow any student to successfully meet the course requirements and achieve a higher grade irrelevant of their prior educational background. The adaptive learning technology utilises retrieval practice as a central pedagogical approach, and it was hypothesised that this would lead to long term learning, when measured in a course in the following year of the students’ degree.

### Theoretical framework

The Resources for Equitable Activation of Chemical Thinking (REACT) was selected as the theoretical framework for this study [11]. Chemistry researchers strive to connect nanoscale objects and mechanisms to macroscale properties and processes. Similarly, chemistry education researchers must strive to connect micro-level investigations of students with chemistry concepts (learning materials and classroom interactions), to macro-level contexts and phenomena that are both shaped by and shape, micro-level objects and processes. For example, attitudes across the chemistry discipline or within institutions shape how students participate within a chemistry class. An instructor’s teaching motives and choice of activities also shape the attitudes of large groups of students. All these external factors shape which students remain in chemistry classrooms and what attitudes therefore the cohort tend to hold. The REACT framework models the learner’s response to the learning environment and interventions (as presented in this study), as well as the way the environment is shaped by the instructor’s behaviours, the institution’s identity, and the cultural ideas of what chemists should know and how they behave.

The REACT framework centres around maximizing learners’ engagement with the chemistry discipline during a particular experience. **Engagement**, broadly, refers to a learner’s focus, participation, and persistence on a given task or experience [12–14]. In chemistry education the goal is for students to engage where they apply both chemistry knowledge and practices, in a way that creates meaning for them [15, 16]. In this study, it is hypothesised that using adaptive learning technology will aid students in creating meaning, personalised to their educational needs.

### Activation Triangle

**Fig 1:**
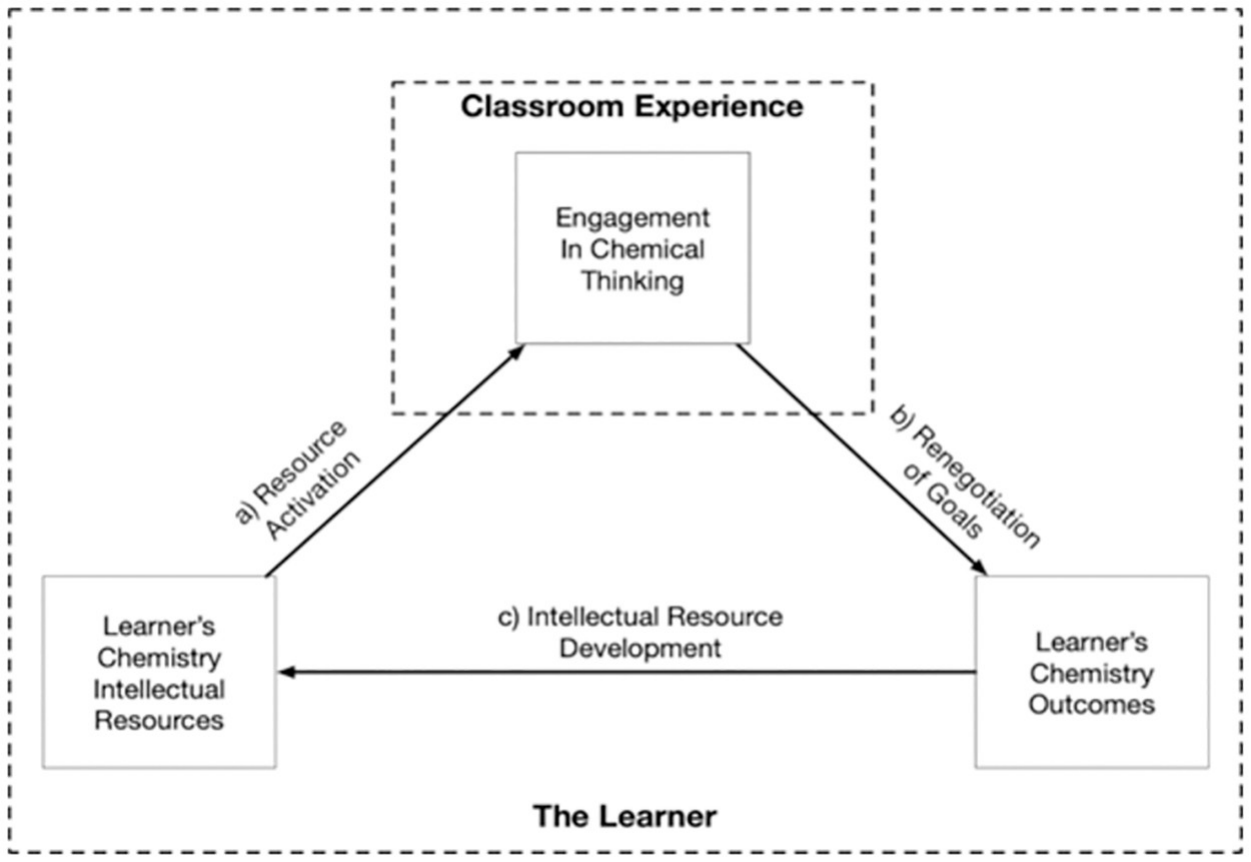
Activation Triangle. A model that shows the recursive relationship between a student’s resources, engagement, and chemistry outcomes.

The Activation Triangle represents the recursive relationship between: 1) a learner’s intellectual resources, 2) their choice to engage in thinking and learning, and 3) a student’s final outcomes and successes (Fig 1) [11]. When presented with a classroom experience in a formal learning environment the student must be willing and able to engage with the classroom material [17]. This process is referred to as **resource activation** (Fig 1 path a). Depending on the level of engagement the student will reflect on whether they have met the goals set personally or by the instructor (goals that don’t necessarily align). It is through this **renegotiation of goals** (Fig 1 path b) that students may choose to engage, or not, in follow up experiences related to the classroom. Finally, it is through continuous positive engagement in the classroom or outside experiences that students will have their **intellectual resources developed** (Fig 1 path c).

From this model, engagement in chemical thinking depends not only on the resources the learner has, but also the design of the experience and learning environment. If students are not able to engage because of the level of intellectual resources they have, it is the instructor’s duty to make changes to the learning environment to activate students’ resources. This leads to the central problem surrounding this research project. How can an instructor, with limited time and resources, help students equitably, despite their differing levels of prior knowledge in chemistry?

### Adaptive learning

Personalised learning refers to the practice of tailoring teaching approaches to the specific needs of each individual student in a class [18]. One specific approach to personalisation is adaptive learning, a pedagogy that utilises technology to dynamically adapt the type, or difficulty, of content to match a learner’s demonstrated ability, or in the REACT framework their intellectual resources [11]. Recently it has been argued that through a combination of automated and instructor interventions, adaptive technologies can accelerate the learning for individual students [19]. This occurs by allowing students the amount of time they need to learn a concept, and by also offering them multiple representations and descriptions of a learning task [20]. While effective educators have been providing this level of personalisation, either explicitly or implicitly, in a face-to-face environment throughout history, the power of technology to achieve this through artificial intelligence can potentially revolutionise this pedagogy at scale.

Adaptive learning has been identified as a key development in the higher education landscape for over six years [21]. Despite the anticipated positive impacts of adaptive learning for providing scalable and equitable education, it has yet to reach its full potential. While there is evidence to support the short-term benefits of adaptive learning on student outcomes in health-related topics [22, 23], previous studies have not probed how adaptive learning meets the fundamental needs of the student, to improve their engagement with chemical thinking (or discipline specific thinking for other fields). Within the chemistry discipline, recently adaptive learning has been developed for a summer preparatory module and as a diagnostic tool for student preparedness in General Chemistry [24, 25]. While engagement with adaptive learning was found to correlate with student success, there were also strong correlations with other measures of student success and preparedness. This suggests a strong selection bias for students who were likely to already succeed in the unit taking additional steps to engage in learning. Despite providing some discrimination between groups of different levels of engagement, there was little evidence that adaptive learning was able to close the gap between students of different levels of starting Intellectual Resources. Critically, the authors noted that a limit of their study was not treating affective domains of student learning, including attitude, motivation, and self-efficacy [25]. The REACT framework provides a lens to understand how these affective domains and the adaptive learning technology can work cooperatively to improve student learning. Beyond the need for empirical evidence, there are additional administrative and governance barriers to implementing adaptive learning in the higher educational sector that must be addressed.

Adaptive learning is often focussed on the economics of learning, by providing students with appropriate tools for study at the times they have available to study. But, as already discussed within the REACT framework, it is not only whether a learner has the time and willingness to learn but tailoring the learning environment to activate their available resources. By dynamically ensuring that these two factors are constantly balanced, then it may be possible to improve students’ engagement with the learning process. This is particularly important in a higher education context, where independent learning is a critical part of the educational experience. Motivation and self-direction are key aspects of protocol for effective deployment of adaptive learning [26]. While it may seem that in the context of independent learning time is nearly limitless and not bound by the number of hours timetabled for classes, it is well established that scarcity due to conflicting demands on a student’s attention still significantly limits a student’s available resources [27, 28]. Therefore, on top of being engaging, effective adaptive learning activities must also be efficient to maintain student perseverance and progress.

## Research questions

**Fig 2:**
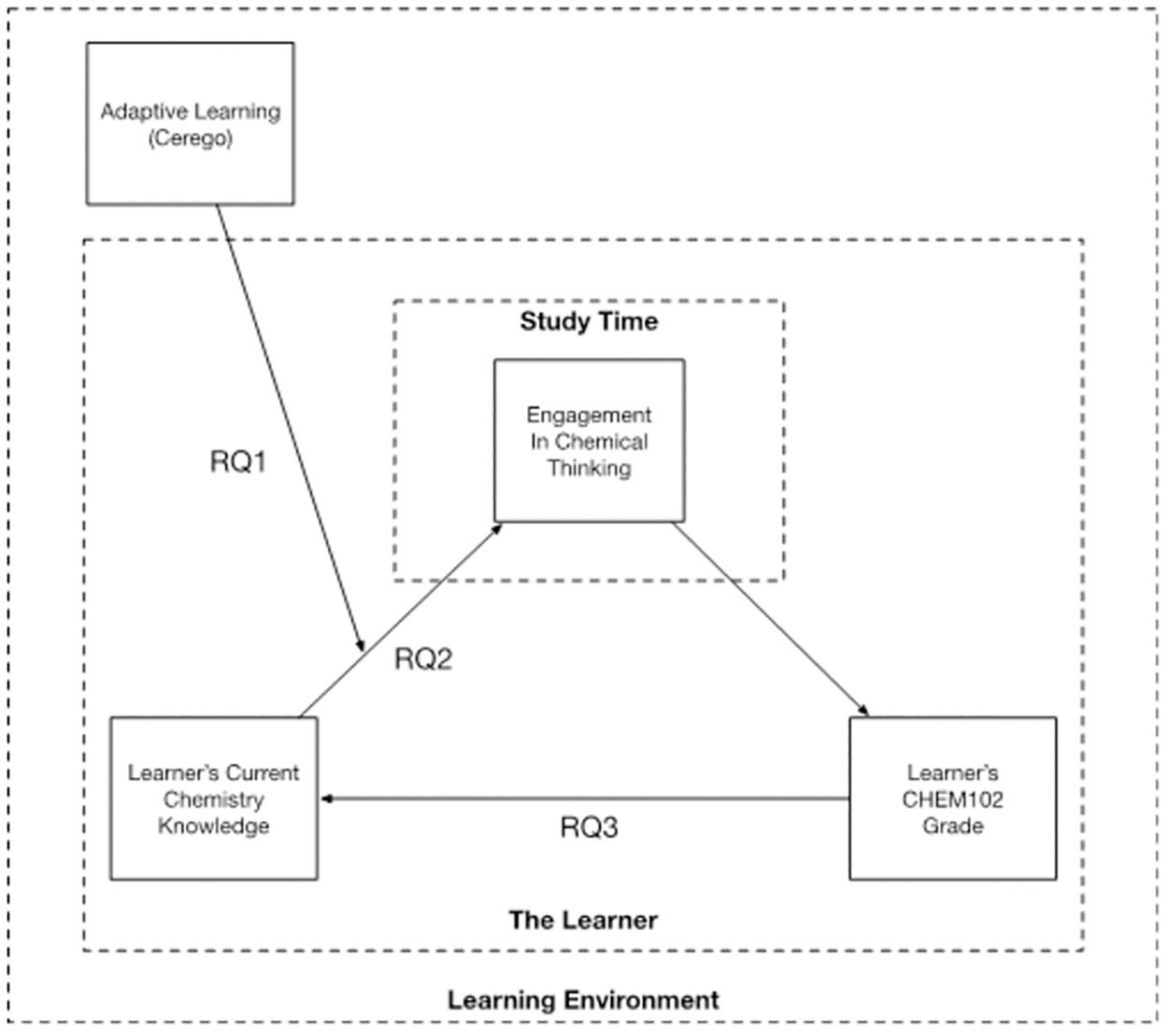
Mapping the research questions to the REACT framework.

This project aimed to deploy an adaptive learning intervention into a first year, introductory chemistry course to aid student learning. Beyond the immediate impacts on student learning, this study aimed to identify how to implement adaptive learning effectively and sustainably by addressing three key research questions (Fig 2).

- RQ1: Is adaptive learning an effective intervention to support learning in chemistry?
- RQ2: Does adaptive learning have differential effects on students based on their current chemistry knowledge?
- RQ3: Are there long-term effects of these interventions on the follow-up chemistry course Organic Chemistry I?

## Methods

### Participants

The participants included in this study were enrolled in foundational chemistry units (*N* = 413) at a large research university in Australia. The authors of the manuscript want to acknowledge the Turrbal and Yugara, as the First Nations owners of the lands where our research took place, lands that were never ceded. We recognise that these lands have always been places of learning, teaching, and research. We pay our respects to their Elders, both past and present. Only 1.4% of the students sampled in this study identified as Aboriginal and/or Torres Strait Islander, and we recognize this is a direct consequence of colonial policies that don’t recognize indigenous ways of knowing and maintain systemic barriers preventing indigenous students to reach their full potential.

Human ethics approval for this project as a low-risk activity was granted by the ethics committee of Queensland University of Technology (1700000102). All research was conducted following ethical and institutional guidelines as outlined in the QUT Manual of Operating Policies and Procedures.

In the current study, data was collected from six cohorts of students across three years (2016–2018) that enrolled in CHEM101 and CHEM102. Overall, 94% of students were government supported domestic students, and 6% were full fee-paying international students. 47% of students in the cohort self-identified as female (binary options only available for reporting at the time of the study). On average, 33% of students were from a low socio-economic background, 15% were linguistically diverse students where English was not their native language, and 2% of students self-identified as having a disability. Furthermore, 67% of students enrolled directly from school, and 33% entered from another pathway, including mature age entry.

### Course structure

First year foundations of chemistry at the university are delivered as two, semester long courses (13 weeks). Students are expected to take both courses simultaneously, not sequentially. CHEM101 covers concepts typical for a general chemistry course, including atomic structure, equilibrium, kinetics, thermodynamics, and electrochemistry (S1 Table in S1 File). CHEM102 is an introductory course focusing on molecular structure and bonding, with concepts from general chemistry and organic chemistry courses being introduced, including nomenclature, functional groups, stereochemistry, and reactivity (S1 Table in S1 File). Students from a range of degrees enrol in these courses, including typically Bachelor of Science, Bachelor of Engineering (Process Engineering) and Bachelor of Medical Laboratory Science. The courses are typically taken within the first year, and concurrently in the same semester, for the Bachelor of Science, though this differs slightly based on degree structure for Engineering and Medical Laboratory Science. ORGO201 is the advanced organic chemistry course and is taken by students in their fourth semester in the Chemistry major of a Bachelor of Science. It expands on the topics covered in CHEM102, with a deeper understanding of the mechanisms of organic reactions, the influence of kinetic and thermodynamic parameters, and advanced spectroscopy (S1 Table in S1 File).

### Adaptive learning intervention

Adaptive learning was delivered to students enrolled in CHEM102 using the commercially available tool, Cerego (https://cerego.com/). All course content delivered through the app was developed by the teaching team, to meet the specific needs of their learners. All enrolled students were invited and provided the tool for free, but participation was voluntary, and it did not contribute to their assessment profile. Students were automatically registered to the tool and invited by email, learning management system announcements (LMS, Blackboard, https://www.blackboard.com/), and discussion in lectures and tutorials explaining how to access and the intended benefits. Online resources explaining how to access and start with the tool were provided in the LMS, as well as allocating time in the first tutorial to get face-to-face assistance.

**Fig 3:**
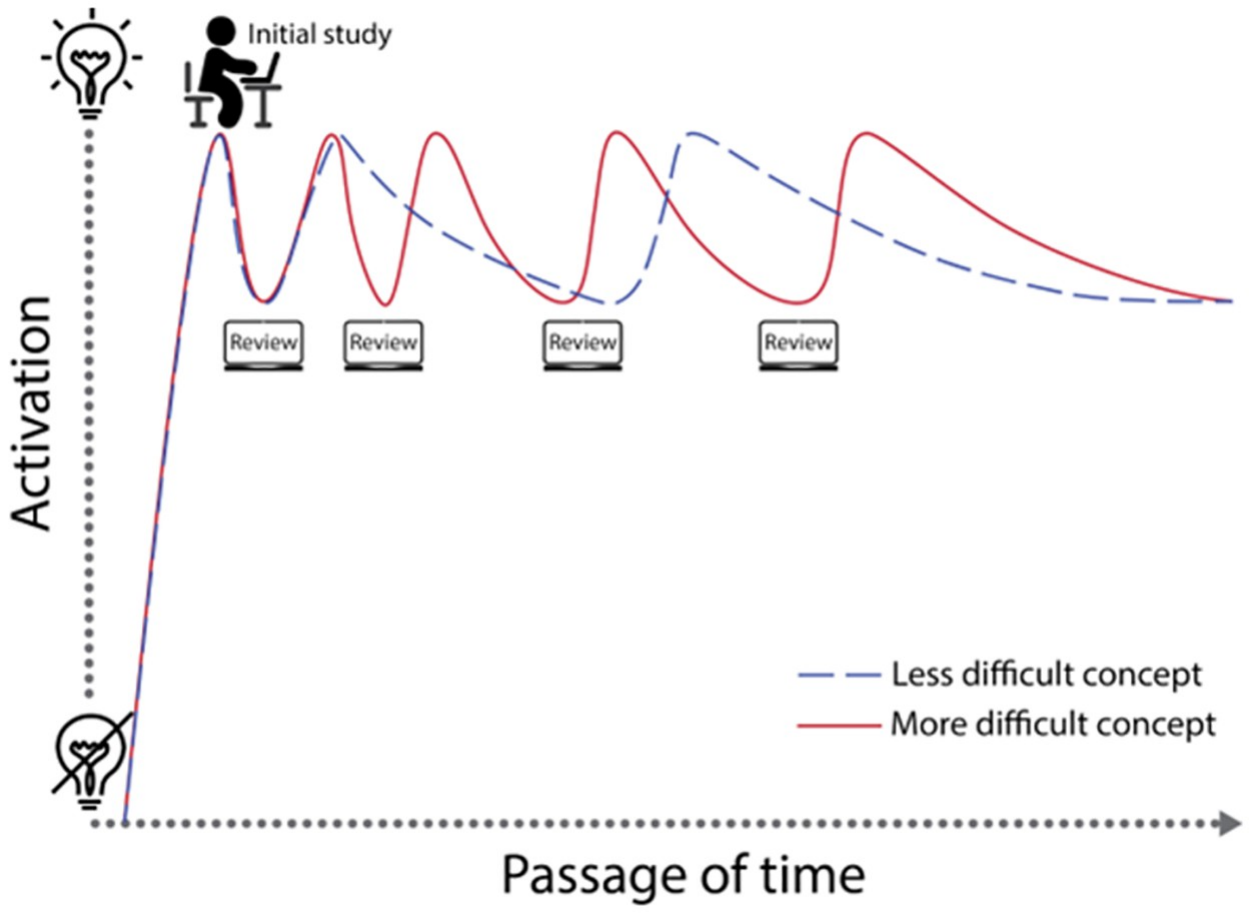
A representation of the learning path in Cerego. Pathways for two different concepts for a single student, showing how the software can adaptively modulate the time of review based on a student’s level of understanding.

The adaptive learning algorithms in Cerego are built upon the learning theories of distributed learning and retrieval practice [29]. For an in-depth discussion of the proprietary learning engine, readers are directed to the whitepaper “Translating Learning Science into Learning Strategy” [30]. In essence the software delivers content to the students in a way that is adaptive to their learning of each individual concept. The content is delivered to the students as a series of practice questions or tasks, which range from multiple choice, fill in the blanks, interactive figures, series, and scenarios. A critical difference between this tool and other homework software, is students are required to revisit past concepts over the course of the study period, with new concepts and revised concepts provided in tandem. The adaptive algorithm times reviews of concepts when they require effort to recall but have a high probability of being recalled successfully (80–90%). By timing this retrieval practice (testing effect) over a longer period (distributed learning), it is intended to enhance long term learning over short term memory recall (Fig 3). In addition, feedback in terms of correct answers or additional information can be provided, which can further enhance the positive benefits of retrieval practice [29]. The tool can be accessed via web browsers, or mobile device applications. The software also provides a visual representation of the learning process to the student through their analytics (similar to the path shown in Fig 3). The analytics also provided to the instructor, allowing for concept or student specific interventions to be designed.

Students were provided with modules to cover a range of concepts in the course, particularly focusing on organic nomenclature, but also covering concepts of organic reactivity and the chemistry of biomolecules. It was anticipated that this would lead to deeper understanding within the unit, but also better retention of concepts for the second-year organic chemistry course, typically taken nine months later.

## Measures

### Outcome measures

In this study two main outcomes were used to represent a measure of achievement: CHEM102 final grade and ORGO201 final grade. It is acknowledged that grades should not be used as the sole representation of a student’s knowledge, skills, or achievement. However, grades as outcomes can be important, as students make decisions about their futures using grades as a benchmark [31]. Furthermore, given the emphasis that universities put on achieving certain grades or standardized scores to be considered "successful", grades can also have an important impact on a student’s perception of their skills and motivation [31, 32]. The assessment profiles for the three course grades used in this study are reported below.

- CHEM101 grade—The assessment profile of this class consisted of an end of semester theory exam (60%), mid semester quiz (20%) and practical laboratory experience (20%)
- CHEM102 grade—The assessment profile of this class consisted of a semester theory exam (50%), quiz assessment and reflective writing (20%), and practical laboratory experience (30%)
- ORGO201 grade—The assessment profile of this class consisted of a semester theory exam worth (45%), research paper on spectroscopy (25%), and practical laboratory experience and report (30%)

The scores reported in this study are the weighted totals for all assessments in a course, expressed as a percentage of the maximum.

In addition to the quantitative data collected from data analytics and assessment, student feedback has been critical in the development of this educational intervention. Feedback from students was collected from end of semester course surveys, dedicated surveys about adaptive learning, and student reflections from CVB102. In the reflective assignment, students are asked to reflect on the educational activities within the subject that had the most influence on their learning experience, with adaptive learning being identified and discussed by some students. The qualitative responses from these sources were collated, and deidentified, before being reviewed for consistent themes, or comments that elaborated on quantitative findings.

### Engagement

Engagement is conceptualized as student’s focus, participation, and persistence on a task [14, 33]. There are four types of engagement that have been shown to be important for chemistry learning: Disciplinary Engagement, Behavioural Engagement, Affective Engagement, and Social Engagement [11, 34, 35]. This study will focus on Disciplinary Engagement & Behavioural Engagement. Disciplinary Engagement refers to learners’ behaviour and knowledge consistent with the discipline of study and its epistemic practices. Behavioural Engagement refers to actions related to participating in the activity regardless of whether that participation is mindful or not [35]. From Cerego’s in-built analytics two values were obtained, time spent using Cerego, and the Set Level of memory permanence. The Set Level is an overall summary of the memory permanence of each concept contained within a module, which is determined using a proprietary algorithm, but is based on the success students have with the learning tasks (S2 Table in S1 File). Therefore, this led to the decision to use time spent on Cerego (App Usage) as a measure of Behavioural Engagement, and "Set Level" as a measure of Disciplinary Engagement. Students were set a learning goal of three within Cerego. This level is expected to take approximately 6 weeks to achieve when cognitively engaged and is intended to lead to long term memory retention that should persist for 3–6 months.

#### App usage

**Fig 4:**
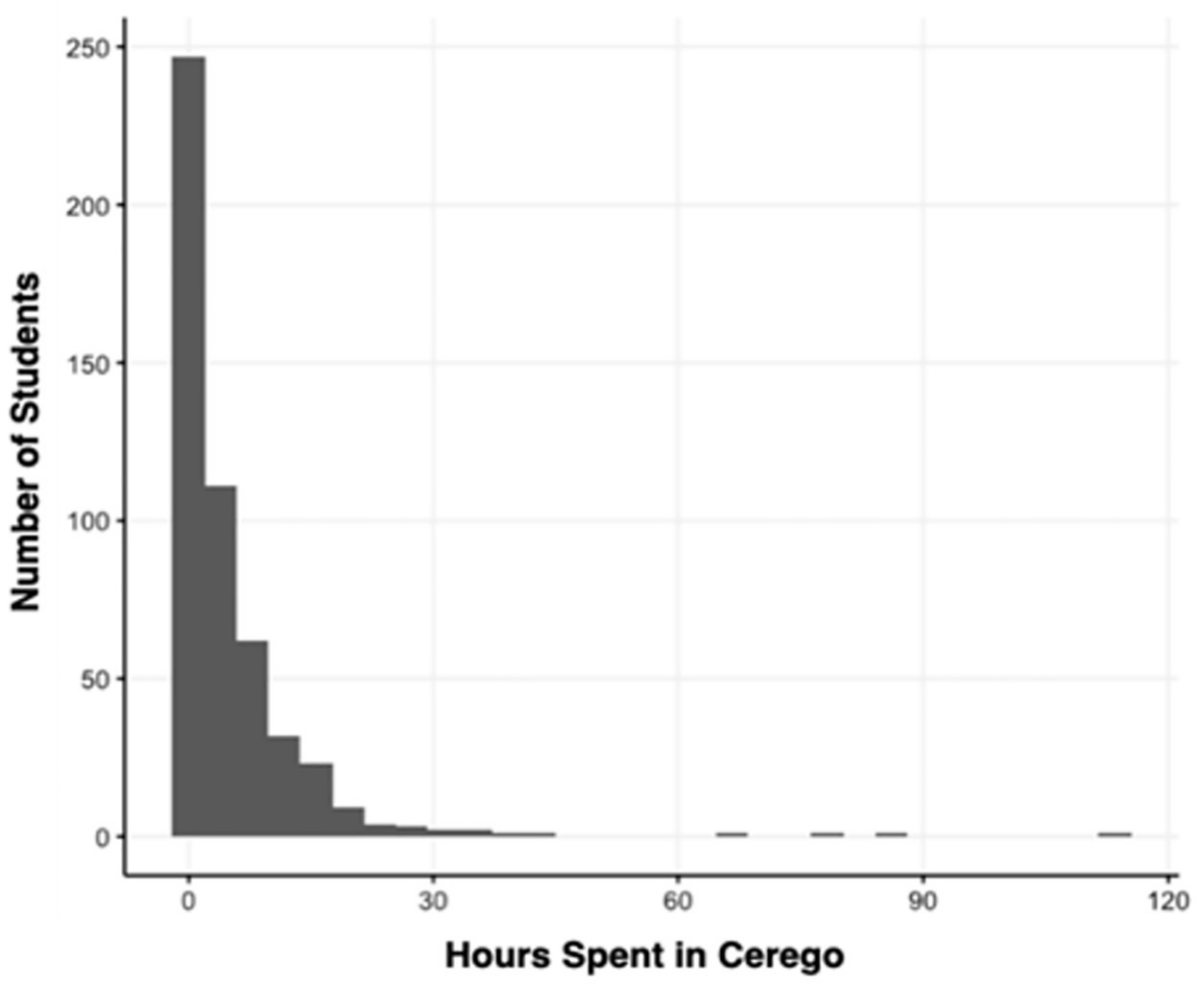
Histogram showing the relationship between hours spent on the Cerego app and number of students.

Fig 4 shows the total number of hours students spent on the Cerego App throughout the semester. The histogram shows a distribution heavily skewed left, with several students showing as outliers (>30 usage hours). Variables that don’t follow a normal distribution run the risk of biasing regression estimates and distorting significance tests [36–38]. For this reason, a variable was created with theoretically meaningful categories:

- Non-takers: Students that never opened the app (N = 123)
- Low Usage: Equivalent of less than 30min per week over 12 weeks (N = 240)
- High Usage: At least the equivalent of 30min a week (N = 141)

#### Disciplinary engagement

**Fig 5:**
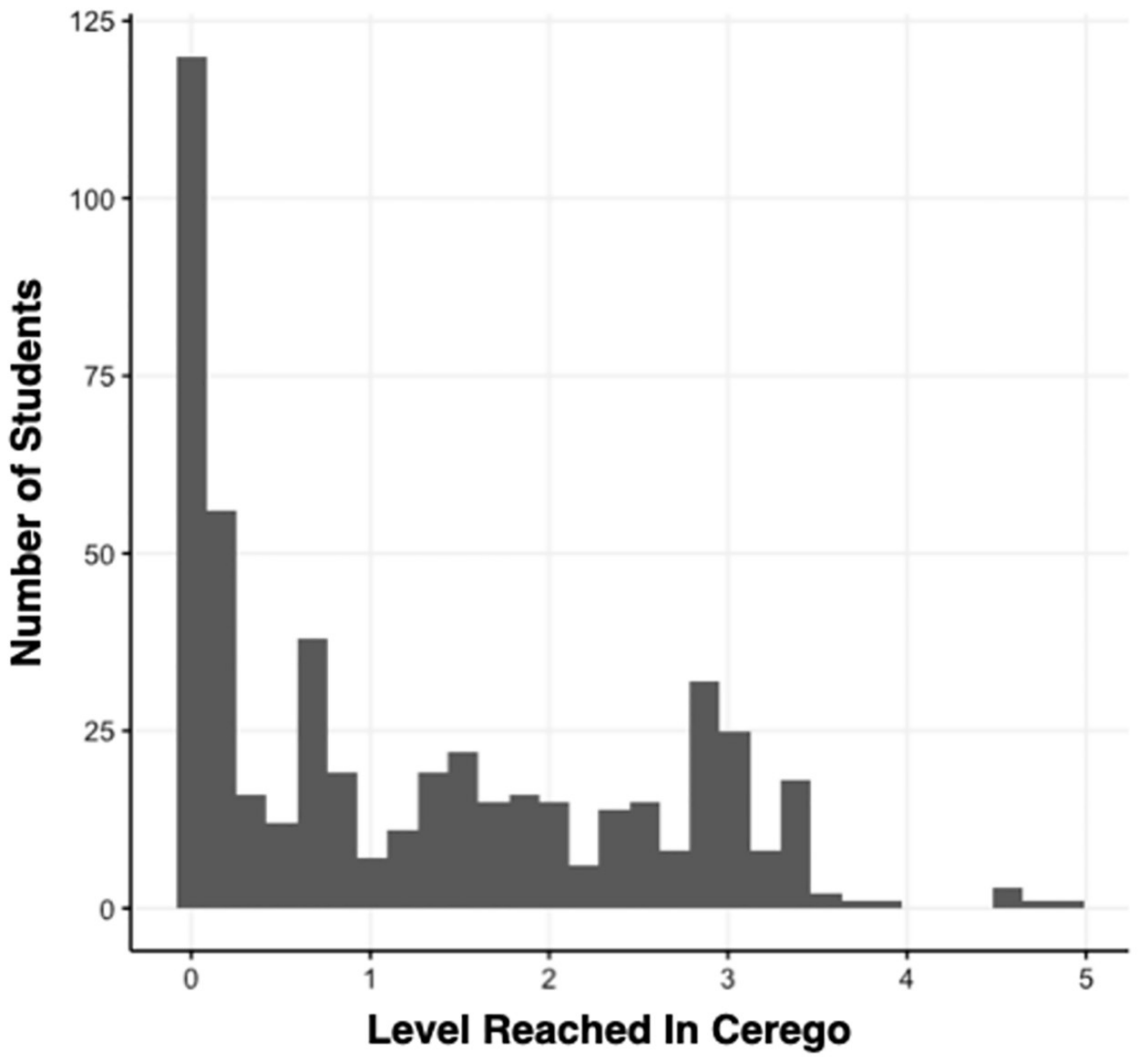
Histogram showing the relationship between level reached on the Cerego app and number of students.

For this the Set Level score provided by Cerego was used. Fig 5 shows the distribution of students across scores. Again, due to the skewed distribution a categorical variable was created.

- Non-takers: Students that never opened the app (N = 123)
- Moderately Engaged: Didn’t reach level 2.5 on the app (N = 266)
- Highly Engaged: Reached above level 2.5 (N = 115)

### Important controls

The use of adaptive learning technology was introduced as a development in scholarship of learning and teaching, aiming to improve engagement and learning for all students in the course. As such it was not designed as a randomised control study, to ensure no students were unfairly impacted. Students voluntarily used Cerego, and participated in any other activities in the study, including surveys or reflections. Students were allowed to opt out of having their results being included in the study. Through the statistical models presented in this study, two variables were considered when accounting and controlling for self-selection in the research study.

- Opt-In—Participating in an intervention was voluntary, and therefore, there may be important motivational or resource differences between students that opt-in versus those who didn’t. Opt-in is a dichotomous variable where "Non-takers" (N = 123) were assigned a code of "0" and everyone that used the app at some point regardless of time or mastery was assigned a "1" (N = 381)
- CHEM101 grade: CHEM101 is the partner course of general chemistry to CHEM102 and did not utilise adaptive learning as part of its curriculum. A student’s grade from CHEM101 has been used to control for differences in motivation and/or educational resources for individual students.

Table 1 shows the correlations between the continuous and categorical variables: 1) r = Pearson correlation, for correlation between two continuous variables; and 2) rpc = polychoric correlation, between continuous and a categorical variable. The coefficients can be interpreted as follows: <0.3 weak relationship, <0.6 medium relationship, >0.6 strong relationship. Overall App Usage and Disciplinary engagement have medium associations with both CHEM101 and CHEM102 grades. The relationship between CHEM101 and CHEM102 is strong, but not strong enough to cause multicollinearity issues.

**Table 1:** Correlations between main variables used in this study.

	GenChem2 Score	Opt-In	App Usage	Disciplinary Engagement
CHEM101 Score	r = 0.59	rpc = 0.42	rpc = 0.34	rpc = 0.39
CHEM102 Score		rpc = 0.56	rpc = 0.48	rpc = 0.55
ORGO201 Score				

To measure the association between categorical variables *Goodman and Kruskal’s tau (GK**τ**) was used [39, 40]*. This measure was chosen as it is the best to handle asymmetric data (where the *N* on each cell is not equally distributed), and relationships where the cross-tabulation has cells with very few or zero cases [41, 42]. Furthermore, this creates the need to analyse both a forward and backwards association (variability in *x* that is explainable by variations in *y* may be very different from the variability in *y* that is explainable by variations in *x*). Table 2 shows the GK***τ*** across all categorical variables. In Table 2 the correlations are read as variability in row variable explains variability in column variable.

**Table 2:** GK*τ* across all categorical variables.

	Opt-In	App Usage	Disciplinary Engagement
Opt-In		0.44	0.48
App Usage	1		0.70
Disciplinary Engagement	1	0.68	

## Results

### Is adaptive learning an effective intervention to support learning in chemistry?

**Fig 6:**
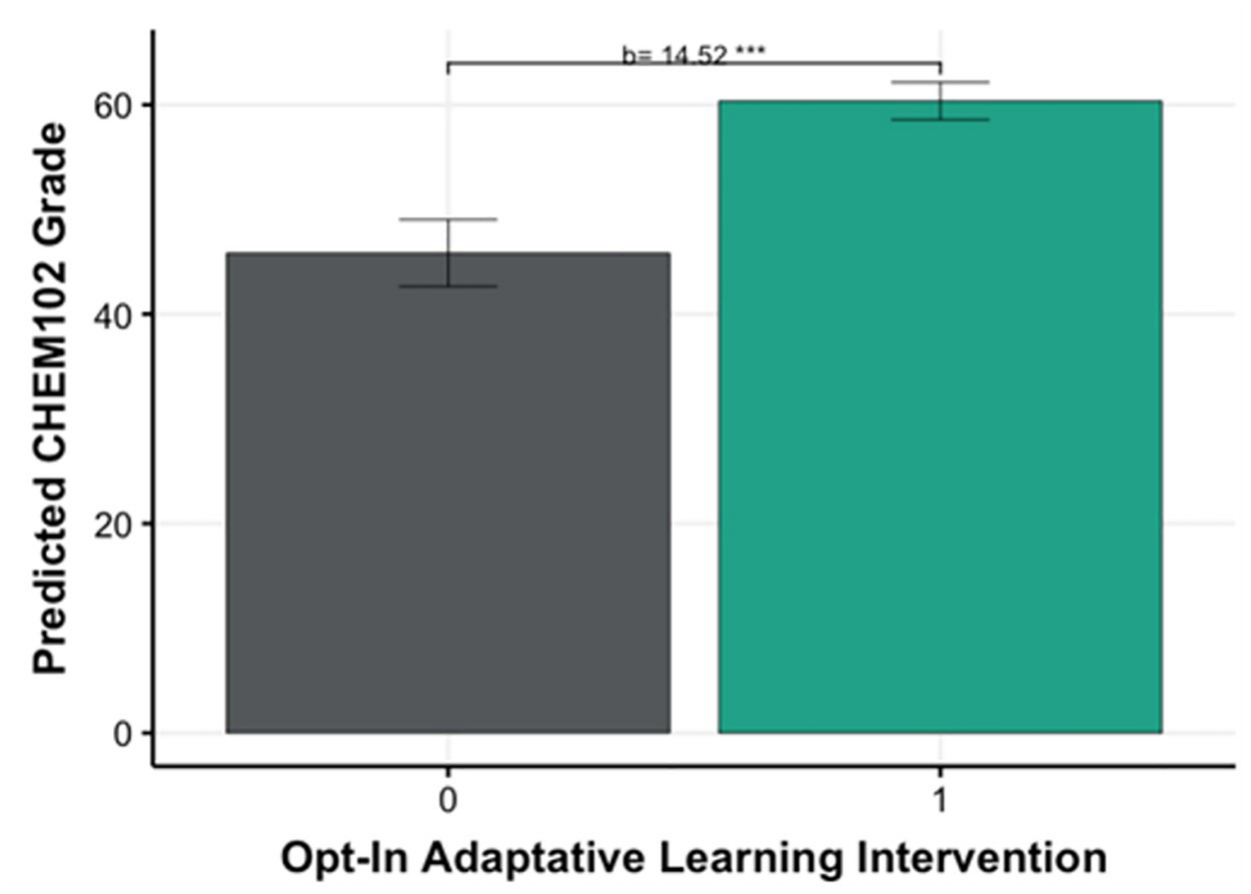
Estimated marginal means (predicted CHEM102 grade) predicted by Disciplinary Engagement, while controlling for CHEM101 grade. (0 = opted out of using adaptive learning, 1 = opted in to using adaptive learning).

There can be important differences between students that choose to use adaptive learning versus those who do not. Therefore, it is important to first understand these differences when looking at the CHEM102 grade as an outcome while controlling for the CHEM101 grade. The regression model in Table 3 shows that on average students that Opted-In (std ***β*** = 0.29, p<0.001), scored 14.52 points out of a 100 in CHEM102, even when accounting for the effect of CHEM101 grade (Std. ***β*** = 0.50, p<0.001). Another way of understanding this effect is by looking at the Std ***β*** of both the Opt-In and CHEM102, where both are considered medium size. That is the strength of the relationship of Opt-In is on average similar to the average CHEM101 grade. Fig 6 shows the difference in predicted CHEM102 score between students that Opt-In and those who didn’t, assuming the same average CHEM101 score (estimated marginal means).

**Table 3:** Effect of opting-in adaptive learning intervention on CHEM102 scores.

	*CHEM*102_*score*_ = *a* + *β*_1_ *CHEM*101_*score*_ + *β*_2_ *OptIn*_(YES=1)_		
	*β*	Std. *β*	p-value
a (Intercept)	18.26		p<0.001
CHEM101 score	0.43	0.50	p<0.001
Opt-In (YES = 1)	14.52	0.29	p<0.001
R^2^	0.42		
*N*	443 students		

Given that the students that Opted-In have different levels of Behavioural and Disciplinary Engagement, it is important to look at the benefit of the relative level of engagement with the intervention, compared to just whether a student chose to opt-in. Practically speaking, Disciplinary Engagement (relies on students progressing to problems of higher difficulty, rather than) rather than spending many hours on low level problems. To test this, we subdivided students that opted-in to the adaptative learning intervention and created a regression model to compare the effects of behavioural engagement (time on the app) to disciplinary engagement (progression on the app to a higher set level).

Table 4 shows the multivariate regression models comparing the effect of App Usage with Disciplinary Engagement. The model shows App Usage does not add any significant information to our understanding of the CHEM102 score. Therefore, subsequent models will only include the Disciplinary Engagement variable.

**Table 4:** Multivariate regression estimates comparing the effect of App Usage with Disciplinary Engagement.

	*CHEM*102_*score*_ = *a* + *β*_1_ *CHEM*101_*score*_ + *β*_1_ *BehavEng* + *β*_2_ *DscpEng*		
	*β*	Std. *β*	p-value
a (Intercept)	35.81		<0.001
CHEM101 score	0.35	0.42	<0.001
Behavioural Engagement (High Usage)	-0.44	-0.01	0.84
Disciplinary Engagement (Highly Engaged)	9.56	0.24	<0.001
R^2^	0.27		
*N*	333		

### Does adaptive learning have differential effects on students based on their current chemistry knowledge?

It is critical to consider potential equity effects this intervention had on the students. It has been shown so far that the intervention had a positive impact on student outcomes as an aggregate, and based on their relative level of engagement. However, it is also important to look at interventions that equalize individual student outcomes and minimize the effect of prior experiences, resources, or knowledge (Intellectual Resources). In doing so it enables any student to maximise their learning potential. To statistically test this a multivariate model was used that included the interaction between CHEM101 grade and Disciplinary Engagement (Set Level). It was hypothesised that if effective, the effect of the intervention on students with lower grades would be larger than on students with higher CHEM101 grades. Table 5 shows the coefficients for this model. Interaction effects of categorical variables are very difficult to interpret on their own. So, at first glance, the negative interaction term in Table 5 might be conflicting. To understand the overall effect of each type of Disciplinary engagement it is useful to consider the effect on individual student grades (Examples are provided in S4 in S1 File). As an example, three students are considered with equivalent incoming CHEM101 grades of 64.16 (the mean value for this sample), with differing levels of Disciplinary Engagement: Student 1) opted-out of the intervention, Student 2) had Moderate Disciplinary Engagement, and Student 3) had High Disciplinary Engagement.

**Table 5:** Multivariate regression estimates for CHEM102 grade for the interaction of Disciplinary Engagement and CHEM101 grade.

	*CHEM*102_*score*_ = *a* + *β*_1_ *CHEM*101_*score*_ + *β*_2_ *DscpEng* + *β*_3_ (*CHEM*101_*score*_)(*DscpEng*)		
	*β*	Std *β*	p-value
a (Intercept)	13.80		
CHEM101 Grade	0.52	0.60	<0.001
Moderately Engaged	21.24	0.49	<0.001
Highly Engaged	33.54	0.65	<0.001
Highly Engaged x CHEM101	-0.20	-0.30	0.036
Moderately Engaged x CHEM101	-0.16	-0.27	0.022
R^2^		0.45	
*N*		443	

**Fig 7:**
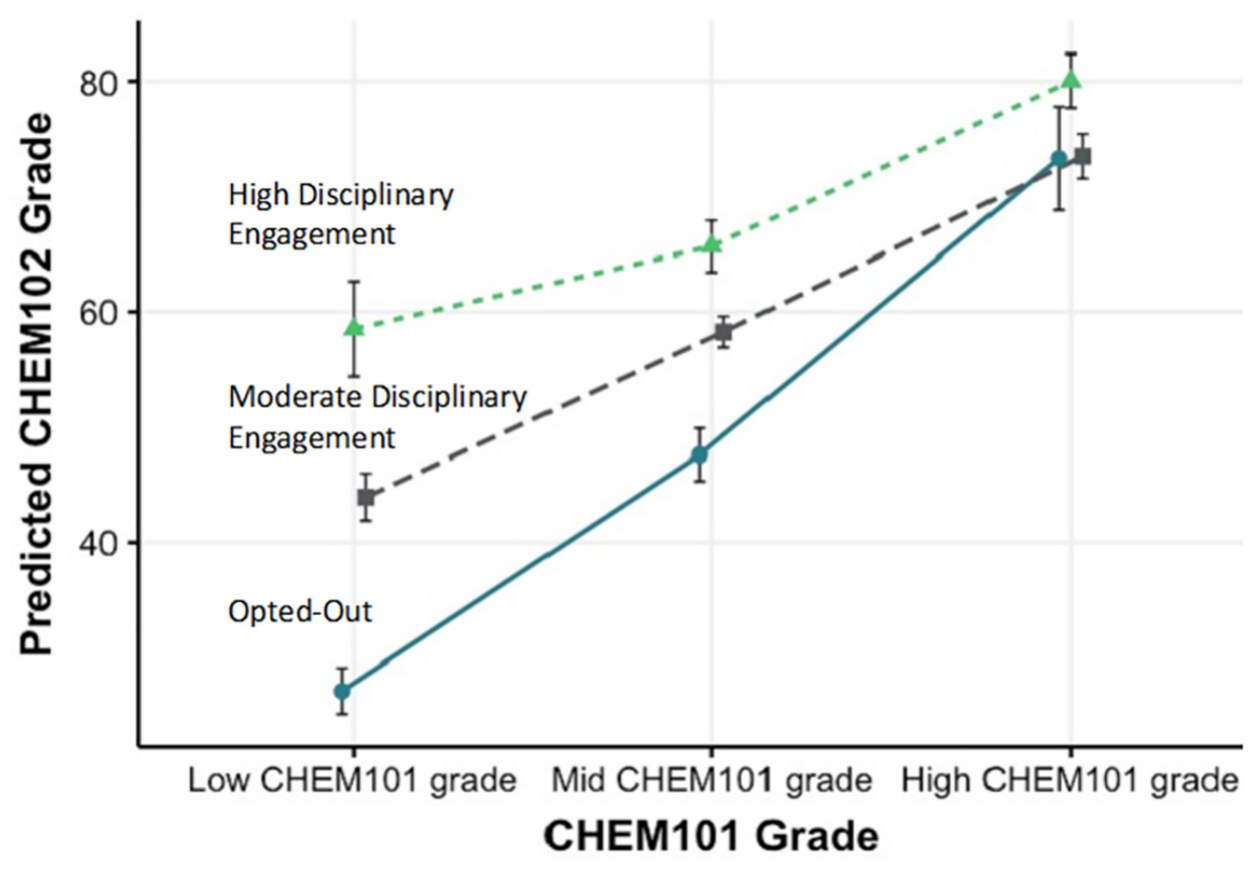
Estimated marginal means for the final grade in CHEM102. Predicted by their incoming CHEM101 grade and their level of engagement with adaptive learning.

Mathematically speaking, the interaction effect is an adjustment to the value of the regression slope by each condition. Practically speaking, for the example of these three students, despite the negative value of the interaction, participating in the intervention has an overall positive average effect on the CHEM102 score (S4 in S1 File. 10.97 points for Moderate Disciplinary Engagement, and 20.75 points for High Disciplinary Engagement). Furthermore, the higher the CHEM101 score, the smaller the impact of the intervention on CHEM102. This is easier to visualize graphically, where Fig 7 shows the estimated marginal means for the final grade in CHEM102, as predicted by their incoming CHEM101 grade and their level of engagement with adaptive learning. To make it easier to visualize we grouped students as: 1) Low CHEM101 grade (less than 60%), 2) Mid CHEM101 grade (61–80%), and 3) High CHEM grade (above 80%). The graph shows that the adaptative learning intervention has a large benefit on students who enter with the lowest CHEM101 grade. This benefit decreases as the CHEM101 score the student comes in with increases. At High CHEM101 scores there is no statistical difference between students no matter whether they participated in the intervention or where Highly or Moderately Engaged. This will be partly an effect of the upper bounds on student grades, where it is not possible for students to exceed a grade of 100%, and therefore cannot improve greatly from their starting point. Overall, this result demonstrates that the benefit from adaptive learning is not dependent upon prior Intellectual Resources (Fig 1).

### Are there long-term effects of these interventions on the follow-up chemistry course Organic Chemistry I?

**Fig 8:**
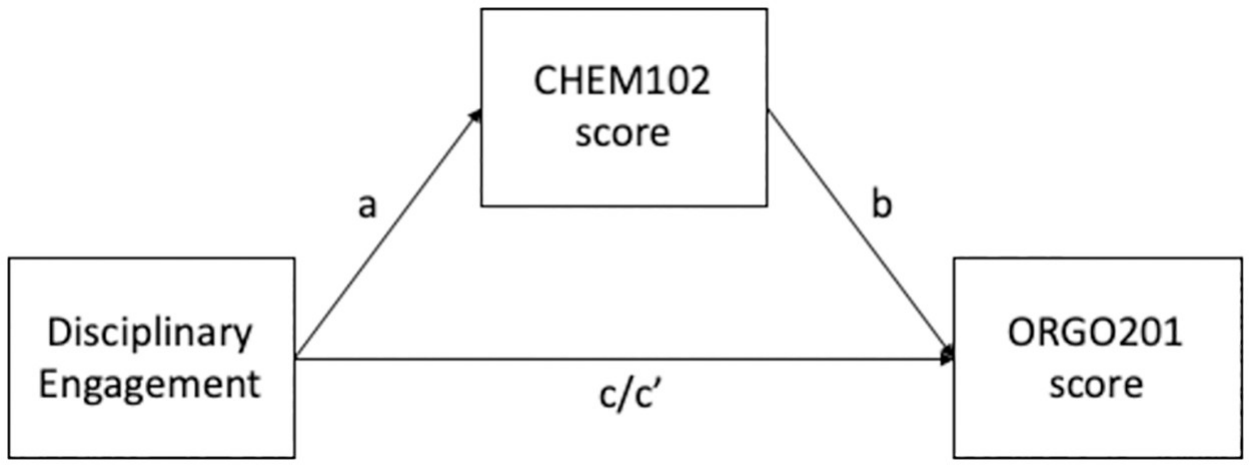
Hypothesized mediation model, testing the impact of adaptative learning intervention on follow up course.

Finally, long-term effects of the intervention on student performance in a related course later in their degree (ORGO201) were investigated. Given that Disciplinary Engagement was an important predictor of CHEM102 grade, this relationship was tested assuming a mediation mechanism (Fig 8). This was done following the procedure proposed by Baron & Kenny (procedure outlined in S5 in S1 File) and the results are presented in Table 6 [43].

**Table 6:** Mediation test for the long-term effect of adaptative intervention usage on the follow-up course.

	Path a		Path b		Path c		Path c’	
Outcome	CHEM102 score		Organic Chemistry 1 Grade		Organic Chemistry 1 Grade		Organic Chemistry 1 Grade	
	Std *β*	p	Std *β*	p	Std *β*	p	Std *β*	p
Opt-In	0.17	0.03			0.16	0.03	0.06	0.94
CHEM101	0.56	<0.001	0.01	0.89	0.34	<0.001	0.006	0.48
CHEM102			0.60	<0.001			0.59	<0.001
R^2^	0.40		0.37		0.07		0.38	

Table 6 shows that there is a benefit in Organic Chemistry I for those students who engaged with adaptive learning (path a). When prior grades are included (Table 6 path c), the correlation between ORGO201 grade and Opt-In to the use of the Adaptative Learning Intervention reaches close to zero and is no longer statistically significant. This hints to a possible mediation mechanism, adaptive learning influences CHEM102 and through that has an effect in the follow up course. Following Baron and Kenny’s procedure there is clear evidence for the hypothesized mediation mechanism. However, given the number of students in this sample (*N* = 97) there aren’t sufficient participants to test this through more rigorous methods like structural equation modelling.

## Discussion

Despite the call for wider use of student-centred approaches to learning in chemistry, there are very few studies focusing on adaptive learning to support students that come into the classroom with different prior experiences and knowledge [5, 9]. In this study, the use of the online adaptive learning tool Cerego, was investigated for its ability to support personalised learning in a foundational chemistry course. Adaptive learning was introduced in this context as an optional blended component of independent study, to support face-to-face classes such as lectures, tutorials, and laboratory practicals. It covered some of the fundamental principles of the course, including chemical nomenclature, isomerism, organic reactions, and the chemistry of biomolecules. It was hypothesised that adaptive learning would have a positive benefit on student engagement and perseverance, provide an equitable learning experience and ultimately lead to long term learning.

To support aggregated results from quantitative analysis, qualitative student feedback can provide further insight into what individual students find engaging about adaptive learning. Feedback was collated from evaluation surveys and a student reflective assignment from the course. Some of the student feedback is presented here to provide evidence and elaboration to support some hypotheses regarding the deeper learning and understanding students may be demonstrating, that is not clear from the quantitative data alone.

The first research question asks if adaptive learning is an effective intervention to support learning in Chemistry? Prior to quantitative data being available on the effect on learning, students were providing clear feedback on the benefits of accessibility and convenience of adaptive learning. They reflected on the simple and easy to use interface and the ability to access Cerego on their mobile devices as advantageous to their engagement, allowing them to study wherever they had the opportunity, such as their commute on public transport. Inspiringly for the teaching team, students’ comments indicated they were redirecting time and resources spent on non-productive activities into learning, demonstrating greater engagement in chemical thinking (Fig 1).

*“It’s a fast and easier way to study*. *[I] love how it can replace Candy Crush*.*”*

*(Survey feedback*, *Semester 1*, *2016)*

To obtain empirical evidence for the benefit of adaptive learning, this study focused on Disciplinary Engagement [11, 34, 35], given that cognitive engagement requires the student to be focused. There is often a methodological tension to try to differentiate between disciplinary engagement and just "going through the motions" [12]. By going through the motions, the student engages in behaviours that are related only to completing the task or that are perceived as those of a "good student" at the surface level (Behavioural Engagement) [13, 14, 44]. This distinction is especially important given we relied on the analytical reporting tools from Cerego. To interrogate Discipline Engagement, the Set Level from Cerego analytics was used as a measure, as it not only considered time on task, but measures success and efficiency in completing tasks, therefore demonstrating focus. In this study it was found that students who cognitively engaged through adaptive learning (reached higher levels in the app) were more likely to perform better in CHEM102 (Std *β*: moderate engagement 0.49, high engagement 0.65). This effect was found to be stronger than for time on task, supporting the hypothesis that adaptive learning can facilitate better engagement in chemical thinking, which in turn enhances student success (Fig 2). Furthermore, student feedback provided insight in how adaptive learning was providing better discipline engagement for individual students, rather than just increasing study time.

*“I have resolved to create a more regimented and spaced-out study plan for future quizzes/examinations*, *in order to learn content with the appropriate depth and facilitate better knowledge retention*. *This strategy has been inspired by the Cerego platform*.*”*

*(Student Reflection*, *Semester 2*, *2018)*

To address the second research question and determine if the benefits from adaptive learning in CHEM102 were based on prior chemistry experience, student performance in the companion subject CHEM101, without adaptive learning, were considered. Both usage of adaptive learning (Std *β* moderate: 0.49, high 0.65) and performance in CHEM101 (Std *β* 0.60) were strong predictors of student grades in CHEM102. But when the two variables were analysed together, the strength of the correlation between CHEM101 and CHEM102 grade decreased with increasing usage of adaptive learning (Fig 7). The benefit was strongest for the group of students who had the lowest CHEM101 grade, and hence the lowest level of prior chemistry knowledge. Students with a low incoming grade and a high level of engagement, were moving from a failing grade to a passing grade and performing on par with students in the middle CHEM101 grade bracket (Fig 7). This supports the hypothesis that adaptive learning can support all students entering the Activation Triangle with varying levels of current chemistry knowledge, and by improving their engagement in chemistry specific thinking, can improve their grade outcomes. More importantly, it does this in an equitable way, and is helping to close the gap between high and low achieving students. Student feedback provides some insight into how adaptive learning can facilitate improved learning of past concepts and proficiency with new material.

*“Cerego’s use of spaced repetition and the testing effect to highlight concepts when they are likely to slip from memory aided not only my ability to retain information from previous weeks but aided the speed at which I would understand new but related topics discussed in following weeks.”*

*(Student Reflection*, *Semester 2*, *2018)*

The final research questions aimed to determine if there were long-term benefits from the usage of adaptive learning? Preliminary investigation into quantifying this effect has found a correlation between usage of adaptive learning and a student’s grade outcomes in a second-year organic chemistry course (Std *β* 0.16). From the data presented here, there is some evidence supporting a mediation effect, though the sample size is not sufficient to support this definitively. Despite the limitations of the current quantitative analysis, the fact that a benefit from adaptive learning can be measured in the grade of a follow up course, which covers many topics not covered in the original adaptive learning intervention, nine to twelve months post intervention, is still encouraging. Further investigation of this benefit and how it may be occurring is needed, looking beyond only grades, but it is hypothesised that student understanding and confidence at the start of the course may facilitate more effective learning later.

The principles of adaptive learning and the REACT framework are never explained to the students explicitly. Yet, through their interactions with the tool, and the learning analytics they are provided with on their learning journey, they appear to learn about these principles. Some students were self-reporting changes to their studies habits and attitudes towards learning after using adaptive learning through Cerego. This provides evidence of additional benefits to long-term learning, that is potentially not discipline specific.

*“After days of using Cerego I was already restructuring my entire study routine to be more akin to the short*, *contained*, *but consistent bursts of study that Cerego provided*.*”*

*(Student Reflection*, *Sem 2*, *2018)*

It appears from this feedback that the fundamental learning science concepts of adaptive learning are noticeable to the students, and therefore it helps them to develop as independent learners. This is exciting as the benefits to the learner could grow beyond the bounds of chemistry discipline specific knowledge and skills, and lead to improvements in other domains of learning.

## Conclusions

### Implications for research

In this study empirical evidence is provided for the benefit of adaptive learning. This is also the first empirical evidence for the Activation Triangle (Fig 1) proposed as part of the REACT Framework, supporting its use as a relevant theoretical framework for understanding STEM education [11]. From the REACT model, the evidence supports that resource activation using adaptive learning can meet the individual needs of the learner, irrelevant of their starting resources. This in turn aids them to engage in chemical thinking, improve their learning outcomes, and ultimately leads to long term intellectual resource development. Beyond the discipline specific engagement, student feedback provides evidence that strategies to think and engage as scientists and tertiary education students more broadly is also enhanced. The evidence presented here shows that adaptive learning can lead to real, meaningful, and lasting improvement to student educational outcomes, irrelevant of their prior experience with chemistry.

### Implications for practice

The mobile and accessible nature of this adaptive learning approach is helping to address the issue of scarcity in students’ time and focus. It allowed them to engage in chemical thinking quickly, easily, and at a time that suits their schedule and needs. It moves beyond adaptive personalisation of the content, to personalising the entire learning experience. It is helping students to identify and increase their engagement with Chemical Thinking, which in turn should improve their Learning Outcomes (REACT, Fig 1). The benefits of accessibility and efficiency are evidenced in feedback from students.

*“Yes well the first week I was like oh man it’s all the time*. *And then you got used to it*, *I was like oh okay*, *I’ll just do it every [day]*, *I would just check it every morning on my way here [to university] and on my way home and that’s more than sufficient*. *It would hardly take the whole transport time*.*"*

*(Student Focus Group Interview*, *Semester 1*, *2018)*

This investigation in adaptive learning was a new experience for the research team, who themselves are educators in the higher education sector and chemistry disciplines. It was a new experience in finding a new piece of educational technology, learning how to integrate it within a curriculum, and navigating the administrative hurdles to allow for adoption. From this experience there are some key learnings for those who are investigating adaptive learning (or other new educational technologies) for their own institution, and how they can best be supported by their institutions (Implications for policy).

It has been widely discussed that one of the barriers to widespread adoption of adaptive learning is a lack of empirical evidence of its benefit to student education [45]. It is hoped that the findings from this report can add to the current body of literature, to help address some of these concerns for adaptive learning [19–21, 46].

### Implications for policy

Despite empirical evidence provided from pilot studies of adaptive learning, there are significant challenges to moving from the pilot scale to a broader institution wide adoption. This project found one of the major challenges can be fragmentation of responsibility and support mechanisms across portfolios for learning and teaching, digital technology solutions, and student support and retention. This can slow down, or completely stall, decision making processes. Another set of barriers are the aversion to high cost or complex digital solutions. This leads to the preference for cheaper, less specific tools, which can require large effort and workhours from teaching staff to make them fit for purpose. Even then, they may not provide the same educational outcomes as a tailor-made product. For institutions to succeed in the space of adaptive and personalised learning, they need to establish good support networks for teaching staff, with a clear and defined development pipeline to adoption. For individual educators, you must ensure the long-term benefits are clearly established and articulated to administration. You will also need to be cognisant of the other agendas for the institution (for example blended learning, equity, student retention), and how your adaptive or personalised learning approach can be integrated to solve these challenges.

Finally, there can be a lack of administrative engagement with faculty into the process of establishing and implementing adaptive learning in their disciplines. Institutions where this has been successful have honoured faculty members as discipline experts in both content knowledge and appropriate pedagogical strategies. By empowering the faculty with a voice in this process, it can increase buy-in by educators and build a culture of innovation. Without this, innovators will rarely succeed in seeing wide-spread adoption of their innovations, irrelevant of the level of success.

### Implications for educators

*“Cerego was also new to me–having a way to effortlessly revise on my phone was something I hadn’t experienced before.”*

*(Student Reflection*, *Sem 2*, *2018)*

This project is the research team’s first foray into adaptive learning, and there have been many lessons learned along the way. A few key lessons are shared here, for those who are also looking to explore adaptive learning in their contexts.

- Framing of adaptive learning is important; it is only one tool at your disposal. It can help you overcome the feeling that you aren’t meeting the needs of all your students, but you can’t simply add it to your class and hope it will meet the needs of all without careful planning and clear communication. You must design how it will fit into your curriculum, how it will interact with other learning activities, and what are its strengths and limitations in your context. Then you must communicate this clearly to your students.
- Adaptive learning is a powerful tool for meeting students where they are currently in their learning journey, without deficit framing. The personalised nature means students at all levels can benefit from the approach, and it does not need to be targeted to certain groups.
- If the adaptive learning tool is well designed and well blended within your learning activities, students will feel it is a value add, rather than another thing on their checklist. This can avoid the need to incentivise the activity as assessment or with points, helping students develop as independent and life-long learners.
- Engage your students in the development of adaptive learning. Ask for their feedback. Ask them to reflect on how it is affecting their learning. Get them to design content, questions, or activities. This project benefited greatly from the feedback and reflections of students, as well as some students who championed the idea, getting involved in the design and evaluation of the tool, or just providing encouragement and inspiration.
- To see the true benefits of adaptive learning takes time. You will need to consider all the points above after each time you start a new cohort with adaptive learning, looking for new ways to maximise student engagement and understanding. This also requires maintenance, as even when you have a system that is working well, if the benefits to students are not clearly and constantly articulated, their engagement can drop off sharply.

## Supporting information

S1 File (DOCX)
