# Supplementary material for: Activating discipline specific thinking with adaptive learning: A digital tool to enhance learning in chemistry
Source: PLoS One. 2022 Nov 15;17(11):e0276086. doi: 10.1371/journal.pone.0276086 (PMC9665387; doi:10.1371/journal.pone.0276086)
Supplement: S1 File — (DOCX) [file pone.0276086.s001.docx]

**Supplementary Information**

**S1 Table. Curriculum for Chemistry Courses**

Curriculum concepts have been related to the ACS Exam Study Guide Concepts for clarity. (<https://uwm.edu/acs-exams/students/student-study-materials/>)

CHEM101

- Atomic Structure
- Structure and Bonding
- Formula Calculations and the Mole
- Stoichiometry
- Solutions and Aqueous Reactions
- Heat and Enthalpy
- States of Matter
- Kinetics
- Equilibrium
- Acids and Bases
- Solubility Equilibria
- Thermodynamics
- Electrochemistry

CHEM102

- Nomenclature
- Electronic Structure and Lewis Structures
- Hybridization, Resonance, Aromaticity
- Stereoisomerism
- Nucleophilic Substitutions and Eliminations
- Electrophilic Additions
- Nucleophilic Addition at Carbonyl Groups
- Nucleophilic Substitution at Carbonyl Groups
- Oxidations and Reductions
- Introduction to Spectroscopy
- Synthesis and Analysis

ORGO201

- Acids and Bases
- Mechanisms: Thermodynamics and Kinetics
- Nucleophilic Substitutions Reactions
- Elimination Reactions
- Addition Reactions: Alkenes and Alkynes
- Addition Reactions: Alcohols and Ethers
- Spectrometry, Spectroscopy, and Spectrophotometry
- Radical Reactions
- Aromatic Reactions
- Carbonyl Chemistry
- Enols and Enolates
- Multistep Synthesis

**S2 Table. Set Level from Cerego**

A summary of the Set Levels based on the proprietary algorithm of Cerego. All details are taken from their website (<https://support.cerego.com/hc/en-us/articles/115004523626-Setting-Goal-Levels-in-Cerego>)

Cerego has 7 Levels of memory permanence (or mastery). The closer your learners stick to the recommended Cerego review schedule, the higher the memory permanence they may achieve.

**Levels of Memory Permanence**

**(Level 0) New** - Learners have studied

**(Level 0) Building** - Learners demonstrated memory (remembered the item after a short delay).

**Level 1** - They’ll have mastery for days. Can be achieved with 1-2 days of practice.

**Level 2** - They’ll have mastery for weeks. Can be achieved in 10 days of spaced practice.

**Level 3** - They’ll have mastery for months. Can be achieved in ~6 weeks of spaced practice.

**Level 4** - They’ll have mastery for years. Can be achieved in ~3 months of spaced practice.

**Mastery** - They’ll have mastery indefinitely. Can be achieved in ~1 year of spaced practice.

**S3 Table. Grading Scale used by courses in this study**

| **Grade** | **Category** | **Weighted Score (100%)** |
| --- | --- | --- |
| 7 | High Distinction | >85% |
| 6 | Distinction | 75% < 85% |
| 5 | Credit | 65% < 75% |
| 4 | Pass | 50% < 65% |
| 3 | Marginal Fail | 40% < 50% |
| 2 | Fail | 25% < 40% |
| 1 | Low Fail | < 25% |

**S4. Multivariate model to test interaction of CHEM101 and Disciplinary Engagement on CHEM102 grade. Three exemplary students, with matched CHEM101 scores (64.16), but different levels of engagement with Adaptive Learning.**

Proposed regression equation

${CHEM102}_{score}=a+\boldsymbol{\beta}_{1}{CHEM101}_{score}+\boldsymbol{\beta}_{2}DscpEng+ \boldsymbol{\beta}_{3}\left( {CHEM101}_{score} \right)\left( DscpEng \right)$ (1)

${CHEM102}_{score}=a+\boldsymbol{\beta}_{1}{CHEM101}_{score}+(\boldsymbol{\beta}_{2}+ \boldsymbol{\beta}_{3}{CHEM101}_{score})(DscpEng)$ (1.1)

${CHEM102}_{score}=a+\boldsymbol{\beta}_{1}(64.16)+(\boldsymbol{\beta}_{2}+ \boldsymbol{\beta}_{3}64.16)(DscpEng)$ (1.1)

For student 1 (using the ***β*** estimates from Table 4:

${CHEM102}_{score}=a+\boldsymbol{\beta}_{1}\left( 64.16 \right)$(1.2 Student One)

${CHEM102}_{score}=13.80+(0.52)\left( 64.16 \right)$(1.3 Student One)

${CHEM102}_{score}=13.80+32.72$(1.4 Student One)

${CHEM102}_{score}=46.52$(1.5 Student One)

For student 2 (using the ***β*** estimates from Table 4:

$a+\boldsymbol{\beta}_{1}{CHEM101}_{score}+\boldsymbol{\beta}_{2}{DscpEng}_{MOD}+ \boldsymbol{\beta}_{3}\left( {CHEM101}_{score} \right)\left( {DscpEng}_{MOD} \right)$(1.2 Student Two)

${CHEM102}_{score}=13.80+(0.52)(64.16)+21.24{DscpEng}_{MOD}+(-0.16)\left( 64.16 \right){DscpEng}_{MOD}$ (1.3 Student Two)

${CHEM102}_{score}=46.52+21.24{DscpEng}_{MOD}-10.26{DscpEng}_{MOD}$ (1.4 Student Two)

${CHEM102}_{score}=46.52+10.97{DscpEng}_{MOD}$ (1.5 Student Two)

${CHEM102}_{score}=57.49$ (1.6 Student Two)

For student 3 (using the ***β*** estimates from Table 4:

$a+\boldsymbol{\beta}_{1}{CHEM101}_{score}+\boldsymbol{\beta}_{2}{DscpEng}_{HIGH}+ \boldsymbol{\beta}_{3}\left( {CHEM101}_{score} \right)\left( {DscpEng}_{HIGH} \right)$(1.2 Student Three)

${CHEM102}_{score}=13.80+(0.52)(64.16)+33.59{DscpEng}_{HIGH}+(-0.20)\left( 64.16 \right){DscpEng}_{HIGH}$ (1.3 Student Three)

${CHEM102}_{score}=46.52+33.59{DscpEng}_{HIGH}-12.83{DscpEng}_{HIGH}$ (1.4 Student Three)

${CHEM102}_{score}=46.52+20.75{DscpEng}_{HIGH}$ (1.5 Student Three)

${CHEM102}_{score}=67.27$ (1.6 Student Three)

**S5. Investigating long term effects of Adaptive Learning on student learning. Relationship between student grades in ORGO201 and Adaptive learning was investigated through mediation mechanism, following the procedure of Baron and Kenny (1986).**

Step 1:  Show that Disciplinary Engagement is correlated with ORGO201 score (Table 5, path c), even when controlling for CHEM101 grade. This step establishes that there is an effect that may be mediated.

Step 2: Show that Disciplinary Engagement is correlated with the mediator CHEM102 score (Table 5 path a).

Step 3:  Show that the mediator CHEM102 score is correlated with ORGO201 score (Table 5 path b).

Step 4:  Finally, to establish the mediation, show that the mediator CHEM102 score is correlated with ORGO201 score even when Disciplinary Engagement is on the model. Furthermore, if the *β of* Disciplinary Engagement is reduced or reaches zero we can say we have evidence of a partial or full mediation (Table 5 path c’).
